# Temporal Dynamics of Event-Related Potentials during Inhibitory Control Characterize Age-Related Neural Compensation

**DOI:** 10.3390/sym13122323

**Published:** 2021-12-04

**Authors:** Elizabeth R. Paitel, Kristy A. Nielson

**Affiliations:** 1 Department of Psychology, Marquette University, Milwaukee, WI 53201, USA; 2 Department of Neurology and the Center for Imaging Research, Medical College of Wisconsin, Wauwatosa, WI 53226, USA

**Keywords:** inhibitory control, executive function, event-related potentials, electroencephalography, N200, P300, cognitive aging, neural recruitment

## Abstract

Aging is accompanied by frontal lobe and non-dominant hemisphere recruitment that supports executive functioning, such as inhibitory control, which is crucial to all cognitive functions. However, the spatio-temporal sequence of processing underlying successful inhibition and how it changes with age is understudied. Thus, we capitalized on the temporal precision of event-related potentials (ERPs) to assess the functional lateralization of N200 (conflict monitoring) and P300 (inhibitory performance evaluation) in young and healthy older adults during comparably performed successful stop-signal inhibition. We additionally used temporal principal components analysis (PCA) to further interrogate the continuous spatio-temporal dynamics underlying N200 and P300 activation for each group. Young adults demonstrated left hemisphere-dominant N200, while older adults demonstrated overall larger amplitudes and right hemisphere dominance. N200 activation was explained by a single PCA factor in both age groups, but with a more anterior scalp distribution in older adults. The P300 amplitudes were larger in the right hemisphere in young, but bilateral in old, with old larger than young in the left hemisphere. P300 was also explained by a single factor in young adults but by two factors in older adults, including distinct parieto-occipital and anterior activation. These findings highlight the differential functional asymmetries of conflict monitoring (N200) and inhibitory evaluation and adaptation (P300) processes and further illuminate unique age-related spatio-temporal recruitment patterns. Older adults demonstrated lateralized recruitment during conflict processing and bilateral recruitment during evaluation and adaptation, with anterior recruitment common to both processes. These fine-grained analyses are critically important for more precise understanding of age-related compensatory activation.

## 1. Introduction

Various cognitive processes, including episodic memory, spatial reasoning, and executive functioning, tend to decline in the course of typical, healthy aging [1-3]. Deficits in executive functioning, particularly the ability to withhold attentional or behavioral responses to irrelevant or interfering stimuli (i.e., inhibitory control; [4]), have received attention as potential mediators of more global cognitive decline [5,6]. However, the temporal sequence of neural activity underlying successful inhibition and, in particular, the effects of age on this sequence, are not understood [7].

Inhibitory control is commonly assessed using go/no-go and stop-signal tasks. In go/no-go paradigms, participants respond to go stimuli (e.g., the letter A) while selectively inhibiting responses to no-go stimuli (e.g., the letter B), where the participant knows in advance which stimulus is to be inhibited (i.e., the letter B always signals inhibition). Thus, successful performance requires an “internal” self-driven response selection process, aided by learning and memory [8,9]. In stop-signal tasks, participants respond to ‘go’ stimuli (e.g., the letter A) unless they are followed by an unpredictable stop-signal. The prepotent response to the ‘go’ stimulus is activated and must be effortfully retracted based on the externally generated stop-signal. This is therefore a better index of response inhibition than no-go [9]. Indeed, our recent study demonstrates the advantages of using the stop-signal vs. no-go task in revealing age- and Alzheimer’s disease risk-related differences in cognitive event-related potentials (ERPs; [10]).

Neuroimaging studies of inhibitory control, typically using functional-MRI (fMRI), have implicated multiple sequential subprocesses involving right inferior frontal gyrus (IFG), insula, and cingulate cortex that are necessary for successful inhibition: interference/conflict resolution, action withholding, and action cancellation [11]. In addition, left pre-SMA and superior parietal gyrus contribute specifically to interference (i.e., conflict) resolution. However, although fMRI is well-equipped to determine which brain regions are active during a given task, the very slow (i.e., seconds-long) impulse response function greatly limits knowledge about the time course of activity in the relevant networks [12]. Temporal precision is particularly important for studying inhibitory control, for which relevant neural activity primarily occurs within the first ~400 ms [7].

In contrast to fMRI, research with temporally precise event-related potentials (ERPs), derived from electroencephalography (EEG), has isolated two key components of inhibition: N200 and P300 [13-15]. First, the N200 is a fronto-central negativity that occurs approximately 150–350 ms following an inhibitory cue. Source analyses have highlighted the IFG and dorsal anterior cingulate as likely generators [7,16-18]. Despite the earlier conceptualization of N200 as reflecting response inhibition *per se* [19-21], more recent research suggests N200 is specifically tied to conflict monitoring and alerting of the need for inhibition *prior to* the motor ‘response’ underlying inhibition [18,22,23]. Indeed, N200 amplitudes during inhibitory control tasks are associated with concurrent increases in theta power, indicative of cognitive processes that precede motor processing [18], and are evident even on trials with conflict resolution that do not require motor inhibition [24].

The second component important in inhibitory control, P300, is a positive-going wave that occurs ~300–500 ms post-inhibitory stimulus. In most tasks, P300 is maximal over parietal electrodes. However, in the context of inhibition, it is often larger over fronto-central sites, such as those corresponding with the precentral gyrus, pre-SMA, IFG, and cingulate cortex (i.e., no-go anteriorization; [25,26]). This activity is specifically linked to response inhibition, performance monitoring and evaluation, and error correction [17,18,23,24,27]. In line with this conceptualization, the inhibitory P300 is associated with increased delta power, which is thought to be associated with motivated attention and performance evaluation [18]. Because the current project aimed to investigate neural activity underlying successful inhibitory control, earlier components (i.e., N100, P200) reflecting more sensory processes were not examined.

The understanding of the neural underpinnings of age-related differences during no-go and stop-signal inhibitory control tasks is relatively limited. This is particularly true for stop-signal tasks, despite their ability to better control for task demand-related activation [7,9]. We recently examined this with ERPs at midline electrodes. Using inhibitory tasks with high and equal level group accuracy, we found older adults had smaller posterior but larger frontal P300 amplitudes and overall larger N200 amplitudes specific to the stop-signal task [10]. Other inhibition studies have also reported larger or comparable frontal-central P300 activity across age groups [28-30], some also with smaller central-posterior P300 in older adults [15,31]. These patterns suggest age-related compensatory frontal recruitment in older adults [32,33], particularly in anterior relative to posterior sites during successful inhibition [34]. These results are consistent with findings from fMRI studies, which show greater frontal than posterior activation, as well as greater bilateral activation (particularly in frontal lobes) in older than in younger adults [32,34-39]. A pair of fMRI studies specifically showed greater activation during successful no-go inhibition in healthy older compared to young adults in left prefrontal and inferior parietal clusters, which was replicable over a year later [35,36]. Such recruitment has been associated with better maintenance of high-level cognitive performance [32,33,40]. Unfortunately, despite the clear importance of lateralized activity, relevant ERP studies have thus far been limited to midline electrodes, with one exception. Hong, Sun, Bengson, and Tong [30] reported elevated frontal N200 and P300 activation in older adults during response inhibition (i.e., no-go) that was particularly right-lateralized.

The temporal precision afforded with ERPs can clarify the time course and mechanisms of age-related compensatory activation during successful inhibition. Whereas fMRI has revealed regions of greater activation, the impulse response function spans a period of seconds, which captures a number of critical inhibition-related processes. EEG and ERPs provide the ability to understand which specific subprocesses are associated with compensatory activation, breaking down the global concept of inhibitory control to assess which types of recruitment (e.g., left hemisphere, right hemisphere, frontal cortex) are involved in conflict monitoring, detection, and resolution (i.e., N200), and which are involved in the evaluation and adaptation of inhibitory performance (i.e., P300). Moreover, despite the conceptualization of inhibition as reliant upon a number of interacting subprocesses [7,11], ERP research has examined inhibitory control by collapsing data across epochs of several hundred milliseconds (i.e., ~100–350 ms for N200, ~300–500+ ms for P300), rather than taking advantage of its unique ability to capture data with millisecond-level precision. Thus, a finer-grained temporal analysis of recruitment during inhibition, along with the inclusion of a wider array of lateral electrodes to examine specific hemisphere differences, might better characterize and disentangle the role of age-related recruitment *within* each of the specific subprocesses of inhibitory control [12,35,37,40,41]. The current study sought to address these gaps. We first analyzed N200 and P300 amplitudes using traditional ERP time windows, comparing young and older adults during a stop-signal task. The groups had comparable accuracy to preclude neural differences due to task difficulty or effort. Age-related delays in N200 and P300 latency are well-established [10,42] and, due to lack of direct relevance to compensatory activation, they were not analyzed in this study. Instead, we performed a follow-up analysis to interrogate the continuous waveform temporal dynamics within each age group, using temporal principal components analysis (PCA) to extract the relevant underlying activation. Based upon existing ERP research with inhibitory control tasks and compensatory models of cognitive aging [32,43], we hypothesized that successful stop-signal inhibition would produce left hemisphere-dominant N200 (conflict monitoring) and right hemisphere-dominant P300 (response inhibition) amplitudes in young adults. We anticipated that older adults would exhibit bilateral activation, specifically attributable to recruitment of the non-dominant hemisphere (i.e., greater right hemisphere N200 and left hemisphere P300 recruitment). We expected these effects to be greatest at frontal and fronto-central electrodes. We further expected that such differences would result in age group differences in the spatio-temporal activation pattern for these components during stop trials, although there was too little published research to drive specific predictions for this follow-up analysis.

## 2. Materials and Methods

### 2.1. Participants

Healthy older adult participants (*n* = 49) were recruited via newspaper advertisements, screened for health by phone, and compensated monetarily. Young adults were recruited from psychology classes offering course credit (*n* = 42). The Mattis Dementia Rating Scale—Second Edition (DRS-2) was used to assure intact cognition in the older adult participants, with a cut-off score of 130/144 for intact status [44-46]. One older adult participant was excluded due to a DRS-2 score below 130, reducing the older sample to 48. The depression subscale of the Brief Symptom Inventory (BSI; average six items scored 0 (none)–4 (severe)) was used to assure normal mood and group comparability. All the participants were right-hand-dominant. However, only successful inhibition trial ERP data were analyzed; notably, no motor response occurred during these trials.

### 2.2. Materials

#### 2.2.1. Stop-Signal Task

The stop-signal task consisted of a serial stream of letters visually presented at a rate of 750 ms per letter with an interstimulus interval of 0 ms. First, in the go condition, the participants were instructed to press the space bar every time the letter “r” or “s” was presented (504 stimuli, 78 targets). This condition served to establish a prepotent response. Thereafter, in the stop condition, participants were instructed to press the space bar when the letter “r” or “s” appears (684 stimuli, 81 targets), except when the stimulus was followed by a red flash (i.e., the stop-signal, *n* = 36; flash duration = 100 ms; stop-signal delays = 125 ms and 200 ms rather than a ‘staircase’ procedure to prevent predictability but also maintain high accuracy; see [10]). The outcome measures included target and inhibitory accuracy; target response time (RT); and stop-signal reaction time (SSRT), which is the latency for the process involved in stopping the motor response, as estimated from the distribution of observed target RTs (i.e., the probability of responding to a stop-signal trial) and the stop-signal delay [8,47,48].

#### 2.2.2. EEG Data Acquisition and ERPs

Continuous EEG data were collected using a 64 electrode Brain Products actiCAP arranged according to the extended International 10–20 System with ground at AFz and reference at FCz. The data were recorded using Neuroscan SynAmps2 with impedances kept under 50 kΩ. The EEG data were recorded in DC mode with a low-pass hardware filter at 100 Hz and a 500 Hz sampling rate using Neuroscan software (Scan 4.5). The continuous EEG data were processed off-line using EEGLAB (Version 14.1.0) software via MATLAB (Version 7.12, The MathWorks, Natick, MA, USA). The data were re-referenced to a common average of all electrodes and filtered using a band-pass filter from 0.5–100 Hz and a notch-filter from 59–61 Hz.

The continuous data were visually inspected, and channels were rejected as necessary to eliminate channel-level artifacts. The data for the rejected channels were interpolated based on an average of surrounding electrodes. Next, an Adaptive Mixture Independent Component Analysis (AMICA [49]) was used to decompose the data into individual components. Components reflecting eye blink, other ocular movements, and muscle contraction were rejected and removed from the data based on visual inspection. These data were then segmented from 100 ms prior to stop-signal presentation (i.e., the red flash) to 1500 ms after stimulus onset for correct trials only. A baseline correction of 100 ms pre-stimulus was applied to all epochs. The epochs were then examined and rejected as appropriate based on visual inspection. The remaining epochs were averaged and an additional low-pass filter at 20 Hz (zero-phase, 4th-order, Butterworth) was applied. The peak amplitude was computed at frontal (F3, F4), frontal-central (FC3, FC4), central (C3, C4), and parietal sites (P3, P4) between the range of 100–350 ms for the N200 component and 300–700 ms for P300. These electrodes were selected based on typical maxima (i.e., P300 central-parietal, N200 frontal-central), and common reports from aging studies of frontal recruitment, particularly in inhibitory tasks [18,34,35,42].

The follow-up analyses using continuous waveform data aimed to determine whether the traditional N200 and P300 time windows (as employed in the primary analyses) effectively reflected single peaks, or a single ‘phase’ of activation, as indicated by those components, or whether multiple activity phases occurred within these windows. In the event of multiple phases, we endeavored to describe the phase sequence and their corresponding spatial distributions (i.e., which sites were active and in which order), and how those sequences differed by age. Thus, grand average waveforms across inhibitory trials were computed in open-source Brainstorm software [50] using the full 64 channel array. This was followed by temporal PCA (Brain Vision Analyzer 2.0), using each time point as a variable [51,52] across the full 64 channel electrode array over the interval of 120–700 ms in young adults, and 175–700 ms in older adults. These temporal windows targeted the group-specific N200–P300 ranges from the traditional analyses (i.e., with a later window for older adults who had later component latencies). Separate analyses by group were also important to allow emergence of factor structure differences (i.e., temporal-spatial profiles), rather than emphasizing factors that were common to both groups. The factor threshold was set at eigenvalue >1.0 and the minimum total variance accounted for of 5%. The resulting factors were then back-projected to display their corresponding scalp topographies. Based on comparisons of the scalp maps with the raw ERP data, a good fit was achieved without rotation. Thus, the initial orthogonal matrix was retained [52]. A secondary independent components analysis (ICA) was used to confirm scalp topography [53-55]. Consistent with the approach by Dien [56,57], each factor time series was then compared with corresponding electrode-level waveforms from the original data matrix. Electrodes loading on the factor were considered those that occur within the factor-related zone on the corresponding scalp map.

#### 2.2.3. Procedure

ERPs during the stop-signal task were collected as part of a larger study. The participants completed two testing sessions, separated by approximately one week, with individualized testing on both occasions. All the EEG data were collected on a single day. The participants were seated in front of a computer following EEG cap placement and were instructed and monitored throughout (with feedback as relevant) such that gross motor movements and speech were minimized to limit noise in the EEG signal. Although motion artifact was eliminated from the data as needed (see Section 2.2.2), motion artifact did not lead to the loss of stop-signal trials. Moreover, motion was not a relevant outcome measure to quantify or compare across groups because the trials of interest were stop-signal trials, which require the withholding of a motor response. The stop-signal task was presented in MATLAB (version 7.12, The MathWorks). The instructions were read aloud as they appeared on the screen, and the participants had the opportunity to ask questions regarding task instructions. Corrective feedback relative to task performance was provided throughout the practice blocks of each task condition, but no feedback was provided during the test blocks. All the procedures were approved by the University’s Institutional Review Board.

## 3. Results

### 3.1. Descriptive Statistics and Excluded Data

Two older adult participants and one young adult participant were excluded from the analyses due to technical issues during the collection of the EEG data. These exclusions resulted in a final sample of 46 older and 41 younger adult participants. The sample demographics are presented in Table 1. Aside from age, the groups were comparable, except that the older group had, on average, one more year of formal education than the young adults, which was statistically significant. The young group could not have studied beyond a baccalaureate degree (i.e., 16 years), and education was range-restricted, limiting the variance. Thus, despite the statistical significance, one additional year of college education was not expected to significantly contribute to group differences in the study outcomes. However, to ensure this was not the case, education was included as a covariate in the primary ERP analyses.

### 3.2. Task Performance Analyses

The task performance data are shown in Table 2. The groups did not significantly differ on task accuracy measures, either in the preliminary go task or during the stop-signal task, for target responses or the withholding of responses (i.e., inhibition). However, as expected, the older adults demonstrated slower responses to targets and a slower SSRT than the young adults.

### 3.3. ERP Analyses

Repeated measures 2 × 2 × 4 ANOVAs, including *Age* (Young, Older), *Hemisphere* (Left, Right), and *Site* (F, FC, C, P) were conducted to assess the N200 and P300 amplitudes (in μV). Tables 3 and 4 summarize these analyses. Education was added as a covariate but did not significantly contribute to either model (all *ps* > 0.07). Greenhouse Geisser correction was applied where appropriate. The primary results of interest included the main effects of *Age* and the interactions of *Age* with *Hemisphere* and/or *Site*. The spatio-temporal dynamics of the continuous waveforms by age group were then examined using temporal PCA for the 64-channel waveforms for each group (see Section 2.2.2).

#### 3.3.1. N200 Component

**Figure 1.**
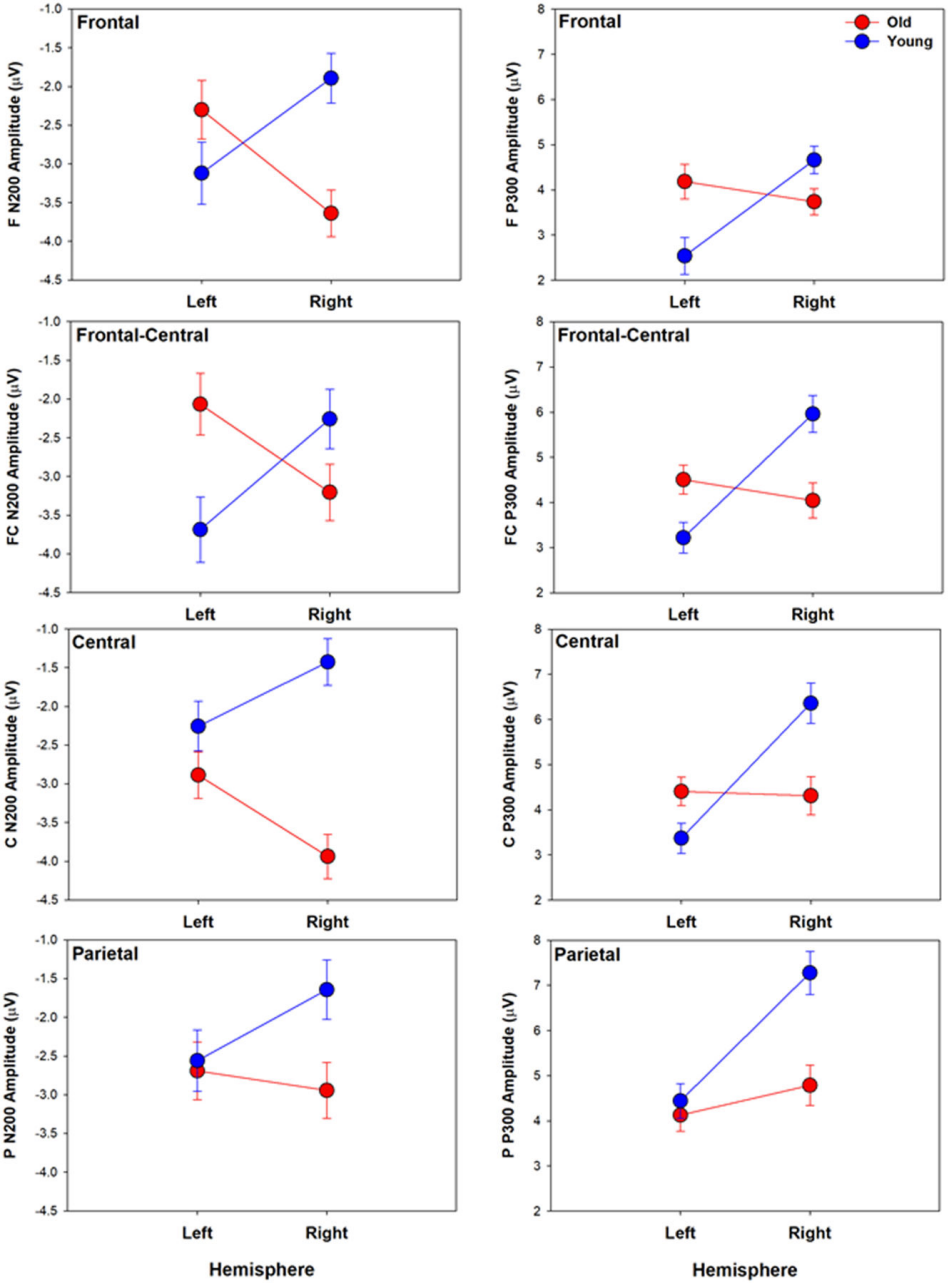
Average ERP amplitude (μV) ± SEM is shown by age group at left (electrode 3) and right (electrode 4) hemisphere sites for frontal through parietal regions (F = frontal; FC = fronto-central; C = central; P = parietal) for N200 (left column) and P300 (right column) components. For N200, a negative-going wave, larger amplitudes are negative (i.e., downward). For P300, a positive-going wave, larger amplitudes are positive (i.e., upward). Corresponding significant group differences are specified in Table 3 and mean and SEM provided in Table 4.

After controlling for years of education, the N200 analyses revealed a significant main effect of *Age* (*F*(1,84) = 4.51, *p* < 0.05, $\eta_{p}^{2}$ = 0.05), with overall larger amplitudes in the older adults (*M*_older_ = −2.96, *M*_younger_ = −2.36). The *Age* by *Hemisphere* (*F*(1,84) = 15.87, *p* < 0.001, $\eta_{p}^{2}$ = 0.16) and *Age* by *Site* (*F*(2.2,181.27) = 7.18, *p* < 0.001, $\eta_{p}^{2}$ = 0.08) interactions were significant. Pairwise comparisons were used to interrogate significant omnibus effects (see Table 3 for corresponding statistics, Table 4 for group means and SEM by factor). The *Age* by *Hemisphere* interaction revealed greater amplitude in older compared to young adults, specifically in the right hemisphere; the older adults demonstrated right hemisphere-dominant N200 amplitudes, while the young adults had larger left hemisphere amplitude (see Table 3). The *Age* by *Site* interaction showed that the older adults’ amplitudes were significantly larger than the young adults’ at central sites, with a non-significant trend at parietal sites (*p* = 0.06). Furthermore, the young adults demonstrated an anterior N200 maximum, while the older adults demonstrated maxima over both frontal and central sites, with less differentiation across the anterior to posterior sites (see Table 3; Figure 1).

#### 3.3.2. P300 Component

After controlling for years of education, the P300 analyses revealed significant Age by Hemisphere (*F*(1,84) = 26.24, *p* < 0.001, $\eta_{p}^{2}$ = 0.24) and Age by Site (*F*(2.2,184.88) = 4.58, *p* < 0.01, $\eta_{p}^{2}$ = 0.05) interactions. Pairwise comparisons were used to interrogate significant omnibus effects (see Table 3 for corresponding statistics, Table 4 for group means and SEM by factor). The interactions revealed right hemisphere-dominant P300 amplitudes in the young adults, with no significant difference between hemispheres in the older adults (i.e., bilateral activation). That is, the young adults demonstrated larger amplitudes than the older adults in the right hemisphere, but the older adults demonstrated larger amplitudes than the young adults in the left hemisphere. Furthermore, the young adults exhibited maximal amplitudes at the parietal site, while the older adults demonstrated a more diffuse pattern of activation that was not significantly different across sites. The age groups significantly differed only in the parietal region, with greater P300 amplitude in young than old (see Table 3; Figure 1).

#### 3.3.3. Post-Hoc Temporal PCA

**Figure 2.**
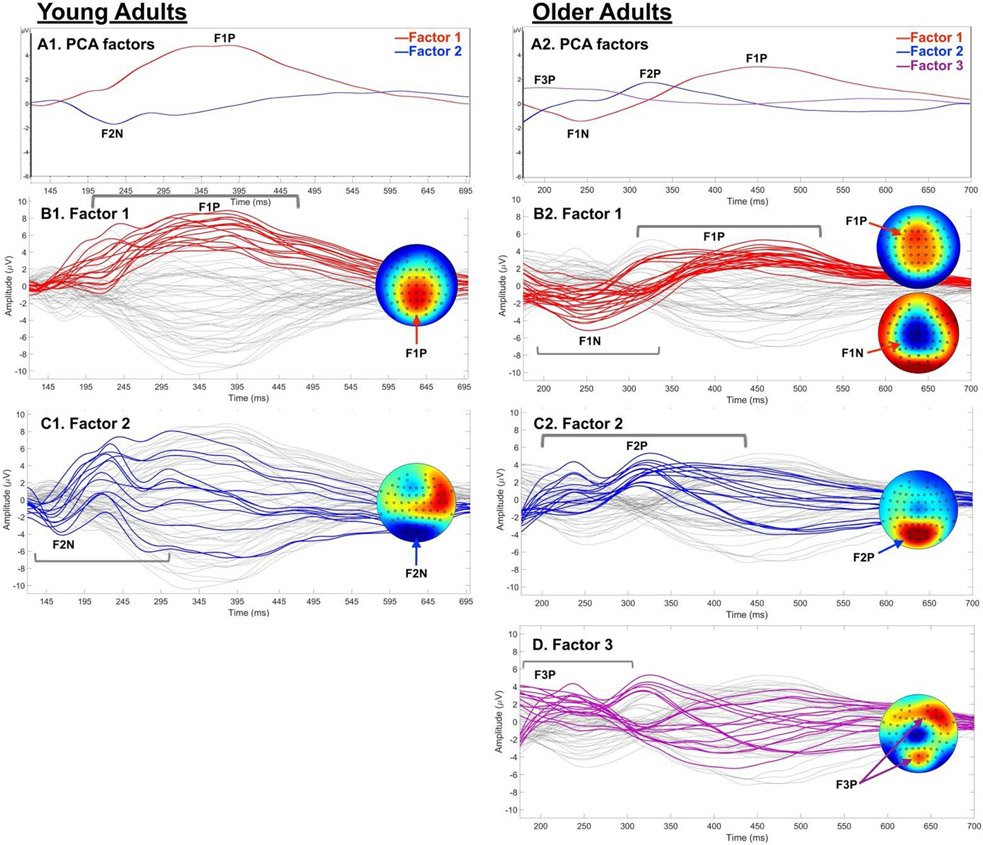
**A**) PCA factor loadings by group. **B-D**) 64-channel grand average ERPs (one tracing/electrode). Electrodes with significant factor loadings shown in color; scalp maps show spatial distributions. Young adults (left): Factor 1) central-parietal positivity (F1P; P300-related; B1); Factor 2 was a parietal-occipital negativity (F2N; N200-related; C1). Older adults (right): Factor 1) two activation phases, with a central negative peak (F1N; N200-related) and a frontal-central positive peak (F1P; P300-related), both of which were diffuse (anterior to posterior extent), with more anterior maximum than Young; Factor 2) two positive parietal-occipital peaks reflecting added visual attention/ processing (F2P); Factor 3) early right frontal positivity (F3P; e.g., P200, sensory-perceptual processing).

Temporal PCA, including all 64 channels, revealed two factors in the young adults (120–700 ms) and three factors in the older adults (175–700 ms). These factors are shown by group in Figure 2; the electrodes that loaded on the factors are listed in Table S1. Notably, the same structure resulted when using a full 0–700 ms range in both groups, but the differentiated windows provided more clarity to the spatio-temporal patterns underlying the factors. In the young adults, Factor 1 captured a single peak of P300-related activity (F1-Y, peak = 380 ms, 87.9% of total variance), with a bilateral central-parietal scalp distribution. Factor 2 captured N200; F2-Y exhibited a single negative peak at 226 ms (7.7% of total variance) and a somewhat left-dominant parietal-occipital distribution. By contrast, Factor 1 in the older adults (F1-O; 74.9% of total variance) included two phases of activation (peaks): (a) a diffuse negative peak (241 ms) with a central maximum that spanned from frontal through parietal sites, thereby effectively capturing the traditional N200 component, but with a broader and more anterior focus than in the younger adults (F2-Y); and (b) a diffuse positive peak (449 ms) corresponing to the P300 component, as in F1-Y, but with a more anterior maximum and an extent ranging from the frontal to the parietal sites. The second factor in the older adults (F2-O; 14.0% of total variance) was characterized by two parietal-occipital positivities (mean = 323ms), suggesting additional visual attentional processing or allocation in the older adults. Finally, in the older adults only, there was also a third factor: F3-O (9.6% of total variance), characterized by an early right frontal positivity (195ms), which is indicative of early sensory-perceptual processing (i.e., P200) rather than being related to the N200 or P300 components, which are the focus of this investigation.

## 4. Discussion

The current study used a high-accuracy stop-signal paradigm to examine age-related differences in lateralized N200 and P300 amplitudes, and to delineate the temporal sequence of compensatory recruitment during successful inhibition. Peak ERP amplitudes were first analyzed within traditional time windows, followed by the examination of continuous spatio-temporal patterns using temporal PCA. The analyses revealed distinct hemispheric patterns, both within and between the young and the healthy, cognitively intact older adults. The young adults exhibited left hemisphere-dominant N200 activation and right hemisphere-dominant P300. By contrast, the older adults exhibited right hemisphere-dominant N200s, which were of larger amplitude overall than in the young adults in the right hemisphere; the left hemisphere amplitudes were comparable between the groups. The older group also demonstrated distinctly bilateral P300 amplitudes, with between-group age effects dependent on the electrode site. Specifically, the older adults demonstrated larger left hemisphere amplitudes, but smaller right hemisphere amplitudes, than the young adults.

A follow-up analysis using temporal PCA allowed for more in-depth characterization of the dynamics underlying traditional N200 and P300 components in these groups. The young and older adults produced different spatio-temporal factor structures. The young adults exhibited two factors, one characterizing P300 with a centro-parietal maximum and one characterizing N200 with a parieto-occipital maximum. The older adults demonstrated a different profile, with three factors. Their first factor included two peaks that captured N200 and P300 with diffuse activation patterns (i.e., anterior to posterior range) and more anterior maxima than in the young adults. The older adults also exhibited a second factor with two parietal-occipital peaks earlier in the P300 window, which likely characterized supplemental visual attention processing; and a third factor with an early right frontal peak representing sensory-perceptual processing. Thus, it was not simply the magnitude or latency of the ERPs that differed in the older adults, but rather, they engaged more diffuse, extensive networks in stop-signal processing. Importantly, this was evident despite the intact and comparable task accuracy in both groups.

### 4.1. N200 Age Group Differences

The N200 activation laterality patterns between the young adults, who were left-dominant, and the older adults, who were right-dominant, during successful inhibition might seem to conflict with the results of fMRI studies of inhibitory control that suggest the right hemisphere is dominant during inhibition [11]. The isolation of the contributing subprocesses, however, has shown that right hemisphere resources are more selectively active during inhibition of the *motor* response, while left hemisphere resources are more selectively active earlier, during pre-motoric conflict detection and monitoring; this is consistent with the role of the N200 component [11,16,18,58]. The young and older adults demonstrated comparable left hemisphere N200 amplitudes, but differed in right hemisphere activation, where the older adults demonstrated significantly larger amplitudes. This supports the interpretation that older adults required additional neural resources to engage pre-motoric conflict processing [17,18,32,59].

Conflict processing that generates N200 during stop-signal tasks includes monitoring for competing (i.e., high conflict) information, specifically the co-activation of the prepotent ‘go’ and inhibitory ‘stop’ responses. Once conflict is detected, N200 may further reflect a sub-conscious alerting or activation of inhibitory mechanisms [18,60,61]. These types of conflict processing are thought to be generated by the dorsal anterior cingulate cortex [7,17,18]. This spatial distribution is consistent with the fronto-central N200 factor in older adults. By contrast, the parieto-occipital distribution in the young adults suggests that visual conflict processing was engaged without need for additional anterior conflict processing to successfully inhibit responding. Combined with the laterality differences between the groups, these patterns suggest that the older adults recruited *both* fronto-parietal and non-dominant hemisphere resources that worked in concert to enable successful conflict monitoring, detection, and resolution. This recruitment thereby contributed to successful task performance despite age-related decline in neural functioning [32,62,63].

To our knowledge, there are only two studies to date of age-related N200 effects using a stop-signal task. One revealed overall larger N200 amplitudes in older compared to young adults at midline electrodes, despite comparable task performance, suggestive of compensation in elders [10], which is consistent with the current findings. The other found no significant age-related differences, which the authors interpreted as age-related compensation [15]. These findings and those from the current study contrast with some previous studies using go/no-go tasks, which overall demonstrated smaller N200 amplitudes in older compared to young adults [64]. However, a 2019 meta-analysis revealed that go/no-go ERP studies also demonstrated poorer overall performance in the older adult groups and, frequently, these studies presented equal numbers of go and no-go trials, which de-emphasized conflict processing invoked by prepotent responding by over-representing the motor inhibition trials relative to traditional paradigms [64]. Moreover, stop-signal tasks provide a number of advantages over no-go tasks, including a better measure of response *inhibition per se* (i.e., vs. response selection) and limiting the role of working memory to allow for more comparable task demand between groups [9]. To effectively capture compensatory recruitment, particularly in a task that is at least moderately demanding, such as inhibitory control, comparable performance between groups is crucial [63]. Thus, the current study, which achieved comparable task performance across groups, likely provided a more accurate reflection of inhibitory control and related compensatory recruitment in aging than was captured by earlier go/no-go studies.

### 4.2. P300 Age Group Differences

As expected, the young adults exhibited right hemisphere dominant P300 amplitudes during successful stop-signal trials, with a central-parietal maximum, evident both in traditional analyses and in their P300-related temporal PCA factor. These findings are consistent with other studies showing right hemisphere-dominant and central-parietal maximal P300 activation [16,59,65]. These patterns coincide with the role of P300 in evaluation and adaptation of motoric response inhibition [7,16,24].

In contrast to young adults, the traditional ERP analyses highlighted distinctly bilateral P300 amplitudes in the older adults. Looking more closely at the bilateral activation in the older adults, the amplitudes in the left hemisphere were larger in the older compared to the young adults, highlighting the recruitment of non-task-dominant hemisphere resources [40]. The right hemisphere amplitudes were significantly smaller in the older group, differing from the N200 findings, where the older adults ‘matched’ the young in the expected regions but recruited additional contralateral resources. For P300, the older adults exhibited insufficiency relative to the young in the expected regions (right hemisphere) and compensated with contralateral recruitment, thereby suggesting the sensitivity of P300 to age-related deficits in neural sufficiency as well as to compensatory recruitment. Specifically, compensatory activation is most consistently evident in elders during low-to-moderate task demand, when their performance is comparable with young groups, while decreased activation becomes evident with high task demand and reduced task performance, which is indicative of depleted neural reserves [63]. P300 was sensitive to this depletion of neural reserve where N200 was not, which is consistent with previous studies showing P300 sensitivity to early neural decline amongst healthy, cognitively intact elders with genetic risk for Alzheimer’s disease [66]. Thus, our findings suggest that the neural mechanisms underlying motor response inhibition and performance evaluation and monitoring via P300 likely decline earlier than N200-related conflict monitoring sources in healthy, typical aging.

The closest comparisons in relevant research to our P300 findings are studies with oddball paradigms, where participants respond to some targets but not to others, which are rarer (i.e., oddballs). The oddball paradigm has often been used because task performance (i.e., task difficulty) is typically comparable between age groups due to the low level of cognitive demand [66]. Importantly, frontal P300 recruitment is evident in older adults in auditory oddball tasks, with compensatory recruitment localized to the precentral and parahippocampal gyri [67]. However, achieving comparable task performance does not necessitate such simplistic paradigms. The stop-signal task can provide comparable performance across groups while also examining the higher-order cognitive processes necessary for maintaining overall cognitive functioning [68] and independent living in older adulthood [69].

### 4.3. Spatio-Temporal Age Group Differences

We performed a post-hoc temporal PCA analysis of the inhibitory trials across time windows designed to include the N200 and P300 peaks in order to examine whether each peak genuinely reflected a single process. In addition, this analysis crucially provided added insight into the multiple subprocesses known to underlie inhibitory control, such as conflict detection, monitoring, and resolution, and inhibitory performance evaluation and adaptation [7,11], and how they differ in young and older adults. Although ERP research on inhibitory control offers the potential to reveal fine-grained temporal subprocesses at millisecond-level resolution, only large time windows of several hundred milliseconds, which are associated with major process divisions (e.g., N200, P300), have been examined. This, taken with a bilateral representation of electrodes, was pursued to better characterize and disentangle age-related differences *within* each of the specific subprocesses of inhibitory control [12,35,37,40,41].

The results of the temporal PCA suggested that a single “process”, or component, effectively represented the N200 and P300 peaks in the young adults. In this case, using traditional mean or peak amplitude metrics is appropriate. By contrast, however, two distinct P300-related factors emerged for the older adults. Given the temporal overlap, traditional amplitude metrics would have been unable parse out these subprocesses, suggesting that a more nuanced spatio-temporal approach may be valuable. Furthermore, these factors clarified the timing and sequence of P300-related processing within the older adult group. First, the two successive early P300 parieto-occipital peaks indicated that there was added allocation of visual attentional resources for P300-related evaluative processes in older adults. Second, the older adults produced a P300 peak that was temporally consistent with the peak produced by the young adults, but the older adults exhibited a more diffuse and anterior scalp distribution. This anterior ‘shift’ was indicative of frontal recruitment to support evaluation and adaptation during inhibitory performance. This effect was also apparent in our prior study, which also featured comparable task accuracy across groups [10]. We suggest that the recruitment of generally bilateral P300 resources along with frontal P300 recruitment and greater early parieto-occipital attention-related activation in older adults contributes to the maintenance of intact inhibitory performance in older age [10,34,70].

Similarly to P300, anterior maximal activity was apparent for the N200 in the older adults, with a more diffuse, bilateral response than in the young adults. Specifically, the older adults exhibited left frontal activation that was comparable to that of the young adults, along with significantly greater right frontal activation. In addition, the older adults exhibited extraneous early right frontal positive activation (Factor 3), as well as early and later parietal-occipital positivities (Factor 2) that were not apparent in the young adults. These were indicative of recruitment to engage supplemental sensory-perceptual and attentional control processes to engage in conflict processing and performance monitoring. Thus, the spatio-temporal analysis revealed multiple specific sub-processes during inhibition that were necessary for successful task performance in older adults that are not clearly characterized by a simpler traditional component analysis. Furthermore, these findings are most consistent with the interpretation of the task-specific recruitment of specific, relevant subprocesses than with a more general, diffuse overactivation or dedifferentiation [32,62,63].

### 4.4. Limitations

The stop-signal task reported in this study was designed to result in high-accuracy performance in both young and older adult participants to control group differences in neural activation based on task demand and to ensure a sufficient number of successful inhibition trials for ERP analysis. Future research using stop-signal paradigms with a more equal distribution of correct and error trials, while maintaining comparable performance across groups [47], would further enable the examination of error trials and provide increased performance variability, which would allow the assessment of ERP * task performance effects, both within and between subjects. Given the high-accuracy and low-demand nature of this task, subjective psychological stress measures were not assessed, but may be of interest in future studies.

The current paper focused specifically on interrogating neural activity underlying executive inhibitory control processes; thus, we focused the analyses on N200 and P300 activation. Future research directed at further parsing out the role of age on earlier sensory processes in such a task may benefit from a similar spatio-temporal investigation, as might studies investigating other complex cognitive processes. Future research will also benefit from the application of more advanced spatial localization procedures to clarify the sources of age-related differences in N200 and P300 processes.

## 5. Conclusions

The current study uniquely reduces the inhibition construct into specific subprocesses to allow the examination of how each is differentially impacted by aging. Compensatory theories of cognitive aging are largely based on fMRI research, which provides information on the scale of seconds. However, relevant neural activity primarily occurs within the first ~400 ms of an inhibitory stimulus [7]. Thus, studies using event-related potentials are uniquely capable of filling the critical gap addressing the temporal sequence underlying compensatory activation, such as is associated with aging. We explored traditional ERP metrics, collapsing activation over several hundred milliseconds, and we also explored continuous waveform activation with all 64 electrodes using a temporal PCA. The two analyses clarified the spatio-temporal dynamics of N200 and P300 roles in inhibitory control and how they differed by age group. In particular, the older adults’ activation was best characterized when including the supplemental analysis, which helped to reveal both the spatial and temporal sequence difference in P300 between the older and young adults. Furthermore, our findings highlighted the importance of examining hemisphere-specific activation patterns, which may be crucial to understanding both the subprocesses that contribute to successful inhibition in healthy young adults, and the maintenance of cognitive function in older adulthood [32,40]. Thus, we encourage the analysis of both anterior to posterior *and* hemispheric patterns of activation by including lateral electrodes in ERP analyses of complex cognitive functions, especially when assessing the contributions of age.

Given the prominent effect of N200 amplitude during our stop-signal task and the unique underlying spatio-temporal patterns between age groups, attention to the specific neural mechanisms underlying conflict processing during inhibitory control may be a particularly important target for research on healthy, normative aging as well as on risk for pathological aging [10,66]. Temporal PCA also revealed spatio-temporal patterns that differentiated age groups during the P300 window. Thus, given the particularly long time window typically used for P300 analyses, finer-grained temporal analysis of the P300 component could be helpful in revealing and characterizing important subprocesses that may be differentially impacted by aging.

## Supplementary Material

Table S1

## Figures and Tables

**Table 1.** Demographics by age group (mean (±SD)).

	Older Adults (*n* = 46)	Young Adults (*n* = 41)
Age (years)	79.63 (4.68)^a^	19.95 (2.74)^a^
Education (years)	14.80 (2.65)^a^	13.77 (1.16)^a^
Sex (% female)	73.91%	73.17%
Dementia Rating Scale-2^nd^ Edition	138.26 (2.88)	--
Brief Symptom Inventory-Depression	0.39 (0.53)	0.56 (0.61)

Note.

a Significant age group difference (older > young), *p* <.05.

**Table 2.** Descriptive statistics for the stop-signal task by group (mean (±SD)).

	Older Adults (n=46)	Young Adults (n = 41)
Go Task (prepotency):		
% Correct Target Trials (PCTT)	99.52 (0.83)	99.50 (1.51)
Target Reaction Time (ms)	678.71 (47.72)^a^	596.26 (39.51)^a^
Stop-Signal Task:		
% Correct Target Trials (PCTT)	98.58 (2.63)	98.16 (2.52)
% Correct Inhibitory Trials (PCIT)	75.00 (11.92)	77.64 (12.79)
Target Reaction Time (ms)	769.72 (63.36)^a^	684.01 (39.31)^a^
Stop-Signal Reaction Time (SSRT) (ms)	541.47 (36.89)^a^	450.59 (44.94)^a^

Note.

a *p* < 0.001.

**Table 3.** Significant group and hemisphere contrast effects from *Age* by *Hemisphere* interactions for N200 and P300 amplitude during successful stop-signal trials (education covaried).

	Group Contrasts					Hemisphere Contrasts			
		Effect	*F*	$\eta_{p}^{2}$			Effect	*F*	$\eta_{p}^{2}$
**N200**	Left	--				Young	L > R**	8.95	0.10
	Right	O > Y***	25.17	0.23		Older	R > L**	7.42	0.08
	Frontal	--				--			
	Fronto-central	--				--			
	Central	O > Y***	31.79	0.28		--			
	Parietal	--				--			
**P300**	left	O > Y*	5.61	0.06		Young	R > L***	47.93	0.37
	Right	Y > O***	20.00	0.19		Older	--		
	Frontal	--				--			
	Fronto-central	--				--			
	Central	--				--			
	Parietal	Y > O**	10.40	0.11		--			

Note: O = older adults; Y = young adults. L = left (electrode 3); R = right (electrode 4).

*** *p* < 0.001

** *p* < 0.01

* *p* < 0.05.

**Table 4.** Mean N200 and P300 amplitude (μV ± SEM), education covaried) during successful stop-signal trials.

Young adults					Older adults			
	N200		P300		N200		P300	
Site	Mean	SEM	Mean	SEM	Mean	SEM	Mean	SEM
F3	−3.12	0.40	2.54	0.41	−2.30	0.38	4.19	0.38
F4	−1.90	0.32	4.66	0.31	−3.64	0.30	3.74	0.29
FC3	−3.69	0.42	3.22	0.34	−2.07	0.40	4.51	0.32
FC4	−2.26	0.39	5.96	0.41	−3.21	0.36	4.05	0.39
C3	−2.26	0.32	3.37	0.33	−2.89	0.30	4.41	0.31
C4	−1.43	0.30	6.36	0.45	−3.94	0.29	4.31	0.43
P3	−2.56	0.40	4.44	0.38	−2.69	0.38	4.13	0.36
P4	−1.64	0.38	7.27	0.48	−2.94	0.36	4.78	0.45

Note: F = frontal; FC = fronto-central; C = central; P = parietal; 3 = left; 4 = right.

## Data Availability

The de-identified data presented in this study are available on request to the corresponding author (K.A.N.). The data presented herein were reported in part in a conference presentation at the European Cognitive Aging Society’s 5th International Conference on Aging and Cognition (E.R.P.).

## References

[1] Salthouse TA When Does Age-Related Cognitive Decline Begin? Neurobiol. Aging 2009, 30, 507–514, doi:10.1016/j.neurobiolaging.2008.09.023. 19231028 PMC2683339

[2] Verhaeghen P; Salthouse TA Meta-Analyses of age–Cognition Relations in Adulthood: Estimates of Linear and Nonlinear Age Effects and Structural Models. Psychol. Bull 1997, 122, 231–249. 9354147 10.1037/0033-2909.122.3.231

[3] Deary IJ; Corley J; Gow AJ; Harris SE; Houlihan LM; Marioni RE; Penke L; Rafnsson SB; Starr JM Age-Associated Cognitive Decline. Br. Med. Bull 2009, 92, 135–152, doi:10.1093/bmb/ldp033. 19776035

[4] Munakata Y; Herd SA; Chatham CH; Depue BE; Banich MT; O’Reilly RC A Unified Framework for Inhibitory Control. Trends Cognit. Sci 2011, 15, 453–459, doi:10.1016/j.tics.2011.07.011. 21889391 PMC3189388

[5] West R; Alain C Age-Related Decline in Inhibitory Control Contributes to the Increased Stroop Effect Observed in Older Adults. Psychophysiology 2000, 37, 179–189, doi:10.1111/1469-8986.3720179. 10731768

[6] Sweeney JA; Rosano C; Berman RA; Luna B Inhibitory Control of Attention Declines More Than Working Memory During Normal Aging. Neurobiol. Aging 2001, 22, 39–47, doi:10.1016/S0197-4580(00)00175-5. 11164275

[7] Pires L; Leitão J; Guerrini C; Simões MR Event-Related Brain Potentials in the Study of Inhibition: Cognitive Control, Source Localization and Age-Related Modulations. Neuropsychol. Rev 2014, 24, 461–490, doi:10.1007/s11065-014-9275-4. 25407470

[8] Votruba KL; Rapport LJ; Vangel SJ Jr; Hanks RA; Lequerica A; Whitman RD; Langenecker S Impulsivity and Traumatic Brain Injury: The Relations among Behavioral Observation, Performance Measures, and Rating Scales. J. Head Trauma Rehabilit 2008, 23, 65–73, doi:10.1196/annals.1379.009. 18362760

[9] Rubia K; Russell T; Overmeyer S; Brammer MJ; Bullmore ET; Sharma T; Simmons A; Williams SC; Giampietro V; Andrew CM Mapping Motor Inhibition: Conjunctive Brain Activations across Different Versions of Go/No-Go and Stop Tasks. Neuroimage 2001, 13, 250–261, doi:10.1006/nimg.2000.0685. 11162266

[10] Elverman KH; Paitel ER; Figueroa CM; McKindles RJ; Nielson KA Event-Related Potentials, Inhibition and Risk for Alzheimer's Disease among Cognitively Intact Elders. J. Alzheimer’s Dis. 2021, 80, 1413–1428, doi:10.3233/JAD-201559. 33682720

[11] Zhang R; Geng X; Lee TM Large-Scale Functional Neural Network Correlates of Response Inhibition: An fMRI Meta-Analysis. Brain Struct. Funct 2017, 222, 3973–3990, doi:10.1007/s00429-017-1443-x. 28551777 PMC5686258

[12] Slotnick SD fMRI Versus ERPs. In Cognitive Neuroscience of Memory; Cambridge University Press: Cambridge, UK, 2017.

[13] Kok A; Ramautar JR; De Ruiter MB; Band GP; Ridderinkhof KR ERP Components Associated with Successful and Unsuccessful Stopping in a Stop-Signal Task. Psychophysiology 2004, 41, 9–20, doi:10.1046/j.1469-8986.2003.00127.x. 14692996

[14] Chevrier AD; Noseworthy MD; Schachar R Dissociation of Response Inhibition and Performance Monitoring in the Stop Signal Task Using Event-Related fMRI. Hum. Brain Mapp 2007, 28, 1347–1358, doi:10.1002/hbm.20355. 17274022 PMC6871417

[15] Hsieh S; Lin Y-C Stopping Ability in Younger and Older Adults: Behavioral and Event-Related Potential. Cognit. Affect. Behav. Neurosci 2017, 17, 348–363, doi:10.3758/s13415-016-0483-7. 27896714

[16] Huster R; Westerhausen R; Pantev C; Konrad C The Role of the Cingulate Cortex as Neural Generator of the N200 and P300 in a Tactile Response Inhibition Task. Hum. Brain Mapp 2010, 31, 1260–1271, doi:10.1002/hbm.20933. 20063362 PMC6871040

[17] Enriquez-Geppert S; Konrad C; Pantev C; Huster RJ Conflict and Inhibition Differentially Affect the N200/P300 Complex in a Combined Go/Nogo and Stop-Signal Task. Neuroimage 2010, 51, 877–887, doi:10.1016/j.neuroimage.2010.02.043. 20188191

[18] Huster RJ; Enriquez-Geppert S; Lavallee CF; Falkenstein M; Herrmann CS Electroencephalography of Response Inhibition Tasks: Functional Networks and Cognitive Contributions. Int. J. Psychophysiol 2013, 87, 217–233, doi:10.1016/j.ijpsycho.2012.08.001. 22906815

[19] Kiefer M; Marzinzik F; Weisbrod M; Scherg M; Spitzer M The Time Course of Brain Activations During Response Inhibition: Evidence from Event-Related Potentials in a Go/No Go task. Neuroreport 1998, 9, 765–770. 9559953 10.1097/00001756-199803090-00037

[20] Falkenstein M; Hoormann J; Hohnsbein J Inhibition-Related ERP Components: Variation with Modality, Age, and Time-on-Task. J. Psychophysiol 2002, 16, 167, doi:10.1027//0269-8803.16.3.167.

[21] Falkenstein M; Hoormann J; Hohnsbein J ERP Components in Go/No-Go Tasks and Their Relation to Inhibition. Acta Psychol. 1999, 101, 267–291, doi:10.1016/S0001-6918(99)00008-6. 10344188

[22] Smith JL; Johnstone SJ; Barry RJ Movement-Related Potentials in the Go/No-Go Task: The P3 Reflects Both Cognitive and Motor Inhibition. Clin. Neurophysiol 2008, 119, 704–714, doi:10.1016/j.clinph.2007.11.042. 18164657

[23] Groom MJ; Cragg L Differential Modulation of the N2 and P3 Event-Related Potentials by Response Conflict and Inhibition. Brain Cognit. 2015, 97, 1–9, doi:10.1016/j.bandc.2015.04.004. 25955278

[24] Huster RJ; Messel MS; Thunberg C; Raud L The P300 as Marker of Inhibitory Control–Fact or Fiction? Cortex 2020, 132, 334–348, doi:10.1016/j.cortex.2020.05.021. 33017748

[25] Fallgatter AJ; Strik WK The NoGo-Anteriorization as a Neurophysiological Standard-Index for Cognitive Response Control. Int. J. Psychophysiol 1999, 32, 233–238, doi:10.1016/S0167-8760(99)00018-5. 10437634

[26] Salisbury DF; Griggs CB; Shenton ME; McCarley RW The NoGo P300 Anteriorization Effect and Response Inhibition. Clin. Neurophysiol 2004, 115, 1550–1558, doi:10.1016/j.clinph.2004.01.028. 15203056 PMC2706017

[27] González-Villar AJ; Bonilla FM; Carrillo-de-la-Peña MT When the Brain Simulates Stopping: Neural Activity Recorded During Real and Imagined Stop-Signal Tasks. Cognit. Affect. Behav. Neurosci 2016, 16, 825–835, doi:10.3758/s13415-016-0434-3. 27160368

[28] Vallesi A Targets and Non-Targets in the Aging Brain: A Go/Nogo Event-Related Potential Study. Neurosci. Lett 2011, 487, 313–317, doi:10.1016/j.neulet.2010.10.046. 20974222

[29] Vallesi A; Stuss DT; McIntosh AR; Picton TW Age-Related Differences in Processing Irrelevant Information: Evidence from Event-Related Potentials. Neuropsychology 2009, 47, 577–586, doi:10.1016/j.neuropsychologia.2008.10.018. 19022270

[30] Hong X; Sun J; Bengson JJ; Tong S Age-Related Spatiotemporal Reorganization During Response Inhibition. Int. J. Psychophysiol 2014, 93, 371–380, doi:10.1016/j.ijpsycho.2014.05.013. 24905017

[31] Pfefferbaum A; Ford JM ERPs to Stimuli Requiring Response Production and Inhibition: Effects of Age, Probability and Visual Noise. Electroencephalogr. Clin. Neurophysiol 1988, 71, 55–63, doi:10.1016/0168-5597(88)90019-6. 2446846

[32] Reuter-Lorenz PA; Park DC How Does It STAC up? Revisiting the Scaffolding Theory of Aging and Cognition. Neuropsychol. Rev 2014, 24, 355–370, doi:10.1007/s11065-014-9270-9. 25143069 PMC4150993

[33] Cabeza R; Anderson ND; Locantore JK; McIntosh AR Aging Gracefully: Compensatory Brain Activity in High-Performing Older Adults. Neuroimage 2002, 17, 1394–1402, doi:10.1006/nimg.2002.1280. 12414279

[34] Davis SW; Dennis NA; Daselaar SM; Fleck MS; Cabeza R Que PASA? The Posterior–Anterior Shift in Aging. Cereb. Cortex 2008, 18, 1201–1209, doi:10.1093/cercor/bhm155. 17925295 PMC2760260

[35] Nielson KA; Langenecker SA; Garavan H Differences in the Functional Neuroanatomy of Inhibitory Control across the Adult Life Span. Psychol. Aging 2002, 17, 56, doi:10.1037/0882-7974.17.1.56. 11931287

[36] Langenecker SA; Nielson KA Frontal Recruitment During Response Inhibition in Older Adults Replicated with fMRI. Neuroimage 2003, 20, 1384–1392, doi:10.1016/S1053-8119(03)00372-0. 14568507

[37] Langenecker SA; Nielson KA; Rao SM fMRI of Healthy Older Adults During Stroop Interference. Neuroimage 2004, 21, 192–200, doi:10.1016/j.neuroimage.2003.08.027. 14741656

[38] Kleerekooper I; van Rooij SJ; van den Wildenberg WP; de Leeuw M; Kahn RS; Vink M The Effect of Aging on Fronto-striatal Reactive and Proactive Inhibitory Control. Neuroimage 2016, 132, 51–58, doi:10.1016/j.neuroimage.2016.02.031. 26899783

[39] Sebastian A; Baldermann C; Feige B; Katzev M; Scheller E; Hellwig B; Lieb K; Weiller C; Tüscher O; Klöppel S Differential Effects of Age on Subcomponents of Response Inhibition. Neurobiol. Aging 2013, 34, 2183–2193, doi:10.1016/j.neurobiolaging.2013.03.013. 23591131

[40] Cabeza R Hemispheric Asymmetry Reduction in Older Adults: The HAROLD Model. Psychol. Aging 2002, 17, 85–100, doi:10.1037/0882-7974.17.1.85. 11931290

[41] Luck SJ An Introduction to the Event-Related Potential Technique; Massachusetts Institute of Technology Press: Cambridge, MA , USA, 2014.

[42] Patel SH; Azzam PN Characterization of N200 and P300: Selected Studies of the Event-Related Potential. Int. J. Med. Sci 2005, 2, 147–154, doi:10.7150/ijms.2.147. 16239953 PMC1252727

[43] Koen JD; Rugg MD Neural Dedifferentiation in the Aging Brain. Trends Cognit. Sci 2019, 23, 547–559, doi:10.1016/j.tics.2019.04.012. 31174975 PMC6635135

[44] Jurica PJ; Leitten CL; Mattis S Dementia Rating Scale-2: DRS-2: Professional Manual; Psychological Assessment Resources, Inc., Lutz, FL, USA: 2001.

[45] Mattis S Dementia Rating Scale: DRS: Professional Manual; Psychological Assessment Resources, Inc., Lutz, FL, USA: 1988.

[46] Monsch AU; Bondi MW; Salmon DP; Butters N; Thal LJ; Hansen LA; Wiederholt WC; Cahn DA; Klauber MR Clinical Validity of the Mattis Dementia Rating Scale in Detecting Dementia of the Alzheimer type: A Double Cross-Validation and Application to a Community-Dwelling Sample. Arch. Neurol 1995, 52, 899–904, doi:10.1001/archneur.1995.00540330081018. 7661728

[47] Logan GD On the Ability to Inhibit Thought and Action: A Users’ Guide to the Stop Signal Paradigm. In Inhibitory Processes in Attention, Memory, and Language; Dagenbach D; Carr TH, Eds.; Academic Press: Cambridge, MA, USA, 1994; pp. 189–239.

[48] Logan GD; Cowan WB On the Ability to Inhibit Thought and Action: A Theory of an Act of Control. Psychol. Rev 1984, 91, 295, doi:10.1037/0033-295X.91.3.295. 24490789

[49] Palmer JA; Makeig S; Kreutz-Delgado K; Rao BD Newton Method for the ICA Mixture Model. 2008 In Proceeding of the IEEE International Conference on Acoustics, Speech and Signal Processing, Las Vegas, NV, USA, 31 March–4 April 2008; IEEE: Piscataway, NJ, USA, 2008; pp. 1805–1808, doi:10.1109/ICASSP.2008.4517982.

[50] Tadel F; Baillet S; Mosher JC; Pantazis D; Leahy RM Brainstorm: A User-Friendly Application for MEG/EEG Analysis. Comput. Intell. Neurosci 2011, doi:10.1155/2011/879716. PMC3090754 21584256

[51] Dien J; Frishkoff GA Principal Components Analysis of Event-Related Potential Datasets. Event-Relat. Potentials Methods Handb 2005, 189–208.

[52] Dien J; Beal DJ; Berg P Optimizing Principal Components Analysis of Event-Related Potentials: Matrix Type, Factor Loading Weighting, Extraction, and Rotations. Clin. Neurophysiol 2005, 116, 1808–1825, doi:10.1016/j.clinph.2004.11.025. 15996897

[53] Makeig S; Debener S; Onton J; Delorme A Mining Event-Related Brain Dynamics. Trends Cognit. Sci 2004, 8, 204–210, doi:10.1016/j.tics.2004.03.008. 15120678

[54] Richards JE Recovering Dipole Sources from Scalp-Recorded Event-Related-Potentials Using Component Analysis: Principal Component Analysis and Independent Component Analysis. Int. J. Psychophysiol 2004, 54, 201–220, doi:10.1016/j.ijpsycho.2004.03.009. 15331212

[55] Debener S; Makeig S; Delorme A; Engel AK What is Novel in the Novelty Oddball Paradigm? Functional Significance of the Novelty P3 Event-Related Potential as Revealed by Independent Component Analysis. Cognit. Brain Res 2005, 22, 309–321, doi:10.1016/j.cogbrainres.2004.09.006. 15722203

[56] Dien J Applying Principal Components Analysis to Event-Related Potentials: A Tutorial. Dev. Neuropsychol 2012, 37, 497–517, doi:10.1080/87565641.2012.697503. 22889342

[57] Dien J The ERP PCA Toolkit: An Open Source Program for Advanced Statistical Analysis of Event-Related Potential Data. J. Neurosci. Methods 2010, 187, 138–145, doi:10.1016/j.jneumeth.2009.12.009. 20035787

[58] Aron AR; Robbins TW; Poldrack RA Inhibition and the Right Inferior Frontal Cortex: One Decade on. Trends Cognit. Sci 2014, 18, 177–185, doi:10.1016/j.tics.2013.12.003. 24440116

[59] Overbye K; Walhovd KB; Fjell AM; Tamnes CK; Huster R Electrophysiological and Behavioral Indices of Cognitive Conflict Processing across Adolescence. Dev. Cognit. Neurosci 2021, 48, doi:10.1016/j.dcn.2021.100929. PMC7868601 33549993

[60] Larson MJ; Clayson PE; Clawson A Making Sense of all the Conflict: A Theoretical Review and Critique of Conflict-Related ERPs. Int. J. Psychophysiol 2014, 93, 283–297, doi:10.1016/j.ijpsycho.2014.06.007. 24950132

[61] Botvinick MM; Braver TS; Barch DM; Carter CS; Cohen JD Conflict Monitoring and Cognitive Control. Psychol. Rev 2001, 108, 624, doi:10.1037/0033-295X.108.3.624. 11488380

[62] Park DC; Reuter-Lorenz P The Adaptive Brain: Aging and Neurocognitive Scaffolding. Annu. Rev. Psychol 2009, 60, 173–196, doi:10.1146/annurev.psych.59.103006.093656. 19035823 PMC3359129

[63] Reuter-Lorenz PA; Cappell KA Neurocognitive Aging and the Compensation Hypothesis. Curr. Dir. Psychol. Sci 2008, 17, 177–182, doi:10.1111/j.1467-8721.2008.00570.x.

[64] Cheng C-H; Tsai H-Y; Cheng H-N The Effect of Age on N2 and P3 Components: A Meta-Analysis of Go/Nogo Tasks. Brain Cognit. 2019, 135, 103574, doi:10.1016/j.bandc.2019.05.012. 31200173

[65] Schmajuk M; Liotti M; Busse L; Woldorff MG Electrophysiological Activity Underlying Inhibitory Control Processes in Normal Adults. Neuropsychologia 2006, 44, 384–395, doi:10.1016/j.neuropsychologia.2005.06.005. 16095637

[66] Paitel ER; Samii MR; Nielson KA A Systematic Review of Cognitive Event-Related Potentials in Mild Cognitive Impairment and Alzheimer’s Disease. Behav. Brain Res 2021, doi:10.1016/j.bbr.2020.112904. 32941881

[67] Van Dinteren R; Huster R; Jongsma M; Kessels R; Arns M Differences in Cortical Sources of the Event-Related P3 Potential between Young and Old Participants Indicate Frontal Compensation. Brain Topogr. 2018, 31, 35–46, doi:10.1007/s10548-016-0542-y. 28101703 PMC5772121

[68] Salthouse TA; Atkinson TM; Berish DE Executive Functioning as a Potential Mediator of Age-Related Cognitive Decline in Normal Adults. J. Exp. Psychol. Gen 2003, 132, 566, doi:10.1037/0096-3445.132.4.566. 14640849

[69] O’Connor MK; Boyle PA Executive Dysfunction in Alzheimer’s Disease. In Research Progress in Alzheimer’s Disease and Dementia; Sun M-K, Ed.; Nova Science Publishers, Inc.:New York, USA, 2007; Volume 1, pp. 25–38.

[70] Reuter E-M; Voelcker-Rehage C; Vieluf S; Lesemann FP; Godde B The P3 Parietal-to-Frontal Shift Relates to Age-Related Slowing in a Selective Attention Task. J. Psychophysiol 2016, doi:10.1027/0269-8803/a000167.

