# Supplementary material for: Temporal Dynamics of Event-Related Potentials during Inhibitory Control Characterize Age-Related Neural Compensation
Source: Symmetry (Basel). Author manuscript; Available in PMC 2022 Aug 2. (PMC9345327; doi:10.3390/sym13122323)
Supplement: Table S1 [file NIHMS1821704-supplement-Table_S1.docx]

**Table S1.** Electrodes loading highly on temporal PCA factors by group from Figure 2.

Note. F = frontal; Fp = frontal polar; AF = anterior frontal; FC = fronto-central; FT = frontal-temporal; C = central; CP = central-parietal; P = parietal; PO = parietal-occipital; O = occipital; odd numbers = left hemisphere; even numbers = right hemisphere.
